# Supplementary material for: Including patient experiences from online sources in guidelines: A natural language processing study on scabies
Source: PLoS One. 2026 Jan 6;21(1):e0339358. doi: 10.1371/journal.pone.0339358 (PMC12773816; doi:10.1371/journal.pone.0339358)
Supplement: Attachment — Submitted filename: PLOS_ONE_Manuscript_Response to reviewers.docx [file pone.0339358.s001.docx]

Overview of the reviewers’ comments and our reactions to them regarding the manuscript titled “Including patient experiences from online sources in guidelines: a natural language processing study on scabies”

| Comments | Reaction  *(Page numbers refer to the tracked manuscript version)* |
| --- | --- |
| . Please ensure that your manuscript meets PLOS ONE's style requirements, including those for file naming. The PLOS ONE style templates can be found at  <https://journals.plos.org/plosone/s/file?id=wjVg/PLOSOne_formatting_sample_main_body.pdf> and  <https://journals.plos.org/plosone/s/file?id=ba62/PLOSOne_formatting_sample_title_authors_affiliations.pdf> | The entire manuscript, including the title page, has been reviewed and revised as needed to align with the provided templates. |
| In your Methods section, please include additional information about your dataset and ensure that you have included a statement specifying whether the collection and analysis method complied with the terms and conditions for the source of the data. | We have added on p. 8 that the forum administrators were briefed on this research and that data was collected and used in compliance with the Terms and Conditions set forth by Dokter.nl. |
| 3. Please provide additional details regarding participant consent. In the ethics statement in the Methods and online submission information, please ensure that you have specified what type you obtained (for instance, written or verbal, and if verbal, how it was documented and witnessed). If your study included minors, state whether you obtained consent from parents or guardians. If the need for consent was waived by the ethics committee, please include this information.  Once you have amended this/these statement(s) in the Methods section of the manuscript, please add the same text to the “Ethics Statement” field of the submission form (via “Edit Submission”).  For additional information about PLOS ONE ethical requirements for human subjects research, please refer to <http://journals.plos.org/plosone/s/submission-guidelines#loc-human-subjects-research>. | The ethics statement specifies that no full formal review by the Ethics Review Committee of the Faculty of Science (BETHCIE) at Vrije Universiteit Amsterdam was required. This determination was based on the fact that data were exclusively obtained from a publicly accessible data source and because no personal and identifiable data (e.g., names, usernames, IP addresses) were collected. Consequently, formal informed consent was not required nor collected. To further safeguard users’ privacy, we do not publish specific comments that could be used to identify the original user and only share comment IDs in our data set (p. 10). |
| 4. Please note that your Data Availability Statement is currently missing the repository name. If your manuscript is accepted for publication, you will be asked to provide these details on a very short timeline. We therefore suggest that you provide this information now, though we will not hold up the peer review process if you are unable. | Data retrieved from the online forum “dokter.nl” are publicly available in the following repository:  <https://doi.org/10.6084/m9.figshare.25722240.v1>  In order to protect the users, comments are shared as comment IDs/links instead of the comment text and can be used to query the complete comment texts from the respective platform. |
| **Reviewer 1** | |
| Integrating an online forum on the topic of interest in order to get information about consumer’s view seems to be a feasible alternative. Using a natural language processing (NLP) seems plausible, although raising some questions about selection bias. Adding some references about this would help to get information about that. Generally, it would be expected that people with very bad experiences or people being very satisfied with a certain intervention would be overrepresented. Are the publications regarding this kind of bias and how to deal with it using internet platforms on medical topics? Would be helpful to add this. | There is indeed a tendency that extreme and especially negative content is overrepresented online, which Roh & Yang (2021) refer to as “extremity and negativity bias.” This has been particularly observed in review and rating platforms where users who are either highly dissatisfied or very satisfied with a particular intervention are more likely to contribute.  However, in the case of online forums, patient engagement is more diverse. Patients use these platforms for a range of purposes—such as seeking disease information, sharing personal experiences, looking for emotional support, or discussing treatments and side effects. Given this broader scope of engagement, we believe the risk of overrepresentation of only extreme or negative experiences is lower compared to platforms that focus primarily on reviews or ratings.  Nonetheless, we fully acknowledge the importance of this bias, and we have added a note on pages 16-17 of the manuscript to highlight that more typical or neutral experiences may be overshadowed by extreme or negative posts. It is also worth noting that, in our case, the focus on patient problems, struggles, and unmet needs may actually make the overrepresentation of extreme experiences somewhat aligned with the aim of our research, as we are particularly interested in the gaps and challenges not yet addressed by the guideline. |
| 255 ff: “the study findings were assessed for relevance and accuracy by these professionals. “ ? were there any criteria? | There do not exist any standard criteria yet for guideline developers to assess such content. As specified on p. 13, professionals assessed the findings broadly based on their accuracy relevance for the guideline. |
| Discussion: you carefully ventilating possible pitfalls and dangers of using public forums like you did. You underline that a human should assess each AI step. How could you identify fake input in dokter.NL ? Adding some aspects mentioned above may help this work to be even more practice relevant. | We have added a specific example how the “human-in-the-loop” approach can be helpful in checking potentially fake posts. Additionally, we have included a reference to an extensive review on detecting, verifying and mitigating online misinformation (p. 16) |
| **Reviewer 2** | |
| 1. Generalizability: While the method is promising, the study focuses solely on scabies and a single Dutch forum. The authors should briefly discuss the potential applicability to other diseases or cultural contexts. | We have specified how these methods could be more broadly applicable beyond scabies and the Dutch forum, including their potential use for other diseases, contexts where they may be particularly valuable, and across different countries (p. 15) |
| 2. Patient engagement: The lack of direct engagement with forum users (e.g., validation of themes) is noted as a limitation. A brief discussion on how to mitigate this in future studies (e.g., involving patient advocates) would strengthen the manuscript. | The reviewer raises, as we believe, an important point. We have extended our discussion on ways to mitigate the lack of direct engagement with forum users in future studies. Specifically, we highlight the potential role of patient representatives in cross-checking experiential knowledge from online sources, as well as the use of additional methods (e.g., surveys) or analyses of other online platforms. We also note the potential of innovative digital approaches to interact directly with patients online and gather feedback on identified themes (p. 16) |
| 3. Technical details: The preprocessing steps (e.g., stop-word removal, stemming) are described, but additional details on model validation (e.g., inter-rater agreement for topic labeling) would enhance reproducibility. | We have added further details on model validation, specifying the types of models tested and how we assessed and selected STM as the model providing the most interpretable topics. |
